# Antibacterial activity of grapefruit peel extracts and green-synthesized silver nanoparticles

**DOI:** 10.14202/vetworld.2021.1330-1341

**Published:** 2021-05-27

**Authors:** Mbarga M. J. Arsène, I. V. Podoprigora, Anyutoulou K. L. Davares, Marouf Razan, M. S. Das, A. N. Senyagin

**Affiliations:** 1 Department of Microbiology and Virology, Institute of Medicine, RUDN University, Moscow, Russia; 2 Department of Food Sciences and Nutrition, National School of Agro-industrial Sciences, University Ngaoundere, Cameroon

**Keywords:** antimicrobials, grapefruit peel, green synthesis, plant extract, silver nanoparticles

## Abstract

**Background and Aim:**

The gradual loss of efficacy of conventional antibiotics is a global issue. Plant material extracts and green-synthesized nanoparticles are among the most promising options to address this problem. Therefore, the aim of this study was to assess the antibacterial properties of aqueous and hydroalcoholic extracts of grapefruit peels as well as their inclusion in green-synthesized silver nanoparticles (AgNPs).

**Materials and Methods:**

Aqueous and hydroalcoholic extracts (80% v/v) were prepared, and the volume and mass yields were determined. The synthesis of AgNPs was done in an eco-friendly manner using AgNO_3_ as a precursor. The nanoparticles were characterized by ultraviolet–vis spectrometry and photon cross-correlation spectroscopy. The antibacterial activity of the extracts was tested on three Gram-positive bacteria (*Staphylococcus aureus* ATCC 6538, clinical *Enterococcus faecalis*, and *S. aureus*) and two Gram-negative bacteria (two clinical *Escherichia coli*) using various concentrations of extracts (100, 50, 25, 12, and 5 mg/mL and 5% dimethyl sulfoxide as negative control). Minimum inhibitory concentration (MIC) and minimum bactericidal concentration (MBC) were determined using the microdilution method. Modulation of cefazoline and ampicillin on resistant *E. coli* and *S. aureus* strains was added to the mixture design response surface methodology with extreme vertices design, with the diameters of inhibition and the fractional inhibitory concentration index as responses and factors, respectively. The antibiotic, the ethanolic extract, and water varied from 0.1 MIC to 0.9 MIC for the first two and from 0 to 0.8 in proportion for the third. Validating the models was done by calculating the absolute average deviation, bias factor, and accuracy factor.

**Results:**

The volume yield of the EE and aqueous extract (AE) was 96.2% and 93.8% (v/v), respectively, whereas their mass yields were 7.84% and 9.41% (m/m), respectively. The synthesized AgNPs were very uniform and homogeneous, and their size was dependent on the concentration of AgNO_3_. The antibacterial activity of the two extracts was dose-dependent, and the largest inhibition diameter was observed for the Gram-positive bacteria (*S. aureus* ATCC 6538; AE, 12; EE, 16), whereas AgNPs had a greater effect on Gram-negative bacteria. The MICs (mg/mL) of the AEs varied from 3.125 (*S. aureus* ATCC 6538) to 12.5 (*E. coli 1* and *E. coli* 2), whereas the MICs of the EEs varied from 1.5625 (*S. aureus* 1, *S. aureus* ATCC 6538, and *E. faecalis*) to 6.25 (*E. coli* 1). There was a significant difference between the MICs of AEs and EEs (p=0.014). The MBCs (mg/mL) of the AEs varied from 12.5 (*S. aureus* ATCC 6538) to 50 (*S. aureus* 1), whereas those of the EEs varied from 6.25 (*S. aureus* 1) to 25 (*E. coli* 1 and *E. faecalis*). Ethanolic grapefruit extracts demonstrated an ability to modulate cefazolin on *E. coli* and *S. aureus* but were completely indifferent to ampicillin on *E. coli*.

**Conclusion:**

Grapefruit peel extracts and their AgNPs exhibit antibacterial properties that can be exploited for the synthesis of new antimicrobials and their EEs may be efficiently used synergistically with other antibiotics against bacteria with intermediate susceptibility.

## Introduction

The year 2020 will be remembered as the year of the pandemic because of Coronavirus Disease and its multi-dimensional catastrophic effects on human lives, with more than 100 million cases and 2.1 million deaths, and its impact on the global economy. This crisis has elicited a unanimous and unprecedented political and social response around the world [1]. However, countries should also pay attention to other silent epidemics, such as resistance to antibiotics, which is constantly growing in medicine, agriculture, and animal breeding. Recent estimates have shown that antibiotic resistance is responsible for 700,000 annual deaths worldwide, 230,000 of which have resulted from multidrug-resistant tuberculosis [2]. The World Health Organization estimates that if nothing is done to address this problem, drug-resistant diseases may cause 10 million deaths each year by 2050 and damage to the economy as catastrophic as the 2008-2009 global financial crisis [2]. Furthermore, economically (linked directly or not to agriculture and animal breeding), antimicrobial resistance could force up to 24 million people into extreme poverty by 2030 [2].

Antibiotic resistance is defined as the ability of bacteria to resist the inhibitory or destructive activity of an antibiotic to which it was initially sensitive [3]. It primarily results from the uncontrolled use of antibacterial drugs both in medicine and in agriculture, which leads to the recurrent exposure of bacteria to sub-lethal doses of antibiotics and results in their adaptation. This adaptation phenomenon results mainly from the enzymatic degradation of antibiotics by bacteria [4], the modification of the antibiotic target [5], change in membrane permeability [6], and alternative metabolic pathways [6]. Interbacterial transmission of antibiotic resistance through horizontal gene transfer (conjugation, transduction, and transformation) has made the situation critical worldwide [3]. To overcome this problem, intensive research has been done in recent years, and the use of plant extracts and nanoparticle has emerged as promising alternatives [6-13]. Medicinal herbs and plant extracts have been successfully used in traditional medicine around the world for millennia [14], and herbal remedies have various advantages because of their availability, fewer reported side effects, cost, high tolerance toward patients, and lack of bacterial resistance [15].

In addition to well-known medicinal plants, researchers have investigated the antibacterial activity of extracts from certain by-products of food consumption and synthesized green nanoparticles to valorize them in the search for new antimicrobials that may overcome antibiotic resistance [16-25]. Therefore, the aim of this study was to assess the antibacterial properties of aqueous and hydroalcoholic extracts of grapefruit peels as well as their inclusion in green-synthesized silver nanoparticles (AgNPs).

## Materials and Methods

### Ethical approval

Ethical approval was not required for this study. All the experiments were performed *in vitro*.

### Study period and location

This study was conducted from October 2020 to January 2021 at the Laboratory of Microbiology and Virology of the Medical Institute of the People’s Friendship University of Russia, Moscow, Russia.

### Material

#### Plant materials

Fresh grapefruits were purchased from a local market in Moscow, washed with distilled water, and dried by wiping with clean papers. The peels were removed and ground with an electric blender into uniform fine particles. Ground peels were directly subjected to the extraction process.

### Bacterial strains

The bacteria used in this study consisted of three Gram-positive bacteria (one clinical *Staphylococcus aureus* and one *Enterococcus faecalis* and a standard strain *S. aureus* ATCC 6538) and two Gram-negative strains (two clinical *Escherichia coli*). All strains were provided by the Department of Microbiology and Virology of the Peoples’ Friendship University of Russia.

### Chemicals and media

Silver nitrate (AgNO_3_) was obtained from PanReac AppliChem, and dimethyl sulfoxide (DMSO) was purchased from BDH Laboratories, VWR International Ltd., USA. The media were procured from HiMedia and all other reagents and chemicals used were of analytical grade.

### Methods

#### Antibiotic sensitivity test

The modified Kirby–Bauer’s disk method described by Mbarga *et al*. [26] was used to study the antibiotic sensitivity of the bacterial strains tested in this study, and the following eight antibiotics disks were used: Amoxicillin, 30 μg/disk; ampicillin, 25 μg/disk; cefazolin, 30 μg/disk; cefazolin/clavulanic acid, 30/10 µg/disk; 30 μg/disk; ceftriaxone, 30 μg/disk; ciprofloxacin, 30 μg/disk; and nitrofurantoin, 200 μg/disk and trimethoprim, and 30 μg/disk.

### Extraction

A maceration technique was performed to extract the grapefruit peel as previously described [27]. Briefly, 30 g of ground grapefruit peel was added to 270 mL of each solvent (distilled water and 80% ethanol) in separate conical flasks. The flasks were covered tightly and shaken at 200 rpm for 24 h at 25°C in a shaking incubator (Heidolph Inkubator 1000 coupled with Heidolph Unimax 1010, Germany). The mixtures were then filtered by vacuum filtration using Whatman filter paper No. 1 and concentrated at 40°C in a rotary evaporator (IKA RV8) equipped with a water bath (IKA HB10) (IKA Werke, Staufen, Germany) and a vacuum pumping unit (IKA MVP10) (IKA Werke, Staufen, Germany). To avoid loss, the extracts were collected when the volumes were small enough, placed into Petri dishes previously weighed, and incubated open at 40°C until complete evaporation. The final dried crude extracts were weighed, the extraction yields were calculated, and 2 g of each extract was dissolved in 20 mL of 5% DMSO to obtain a concentration of 100 mg/mL. The extracts were sterilized by microfiltration (0.22 mm) and stored as a stock solution at 4°C.

### Screening of antibacterial activity of the extracts

The stock solutions were diluted to obtain concentrations of 5, 25, and 50 mg/mL, and the well diffusion method on Mueller-Hinton Agar (MHA) was employed to test the antibacterial activity of the stock solutions and dilutions on overnight cultures of *E. coli* 1, *E coli* 2, *S. aureus* 1, and *S. aureus* ATCC 6538 adjusted to McFarland 0.5 (corresponding to approximately 1×10^8^ cfu/mL). The 5% DMSO was used as a negative control, and the results for the inhibition diameters represented the mean of three repetitions.

### Green synthesis of AgNPs

AgNPs were green-synthesized according to the method described by Scolaro *et al*. [11] with slight modification. Briefly, 1 mL of the aqueous extract (AE) was added to 9 mL of freshly prepared AgNO_3_ at different concentrations (0, 0.625, 1, 1.25, 2.5, 5, and 10 mM). The mixture was shaken in the dark at 60°C and 200 rpm. The color change of the solution from pale yellow to reddish-brown after 30 min indicated the reduction of Ag^+^ ions to Ag^0^ nanoparticles.

### Characterization of green-synthetized AgNPs

After visual observation, the biosynthesis of the AgNPs in a solution was monitored by measuring the ultraviolet (UV)–vis spectra of the reaction mixture. UV–vis spectra were recorded on a PerkinElmer Lambda 950 spectrophotometer from 35 to 800 nm at a resolution of 1 nm. The solution without extract was used as a blank. Particle sizes were analyzed with photon cross-correlation spectroscopy (Sympatec GmbH, Clausthal, Germany). A Nanophox instrument with UVette cuvettes (routine pack, Sympatec GmbH) was used. Single samples were measured 3 times at 25°C. Data from the unique measurements were integrated to produce a single distribution with the PCCS Windox 5 software (Sympatec GmbH) and the size distributions were obtained using the non-negative least squares algorithm. Standard latex samples (20 ± 2 nm) (Sympatec GmbH) and blank samples were analyzed before the measurements to ensure a high accuracy of the measurements.

### Antibacterial activity of AgNPs

The AgNPs solutions prepared with different concentrations of AgNO_3_ were centrifuged at 12,000 rpm for 45 min, and the resulting pellets were washed twice with distilled water followed by ethanol. The pellets were dried at room temperature (25°C), and 0.15 mg was dissolved in 1.5 mL of distilled water to obtain a concentration of 100 mg/mL. The mixtures were sterilized by microfiltration, and the antibacterial activity was determined as described above for plant extracts.

### Determination of minimum inhibitory concentration (MIC) and minimum bactericidal concentration (MBC)

**Figure-1:**
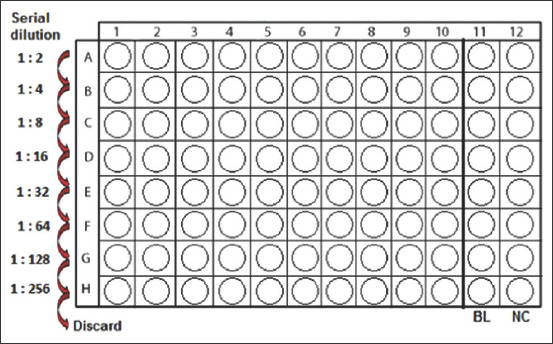
Serial dilution process in microbroth dilution method [28].

The MIC of the extracts and green-synthesized AgNPs (synthesized with 1 mM of AgNO_3_) on the five bacteria tested was determined using the microbroth dilution method as previously described [28]. Briefly, all stock solutions described above were subject to serial twofold dilution in Brain Heart Infusion broth sterile U-bottom 96-well microplates (Figure-1). Next, 100μL of broth was added to all the wells of the plates, and 100 μL of extract (100 mg/mL) or AgNPs (100 mg/mL) was added to the first row. Then, 100 μL of 5% DMSO was added to columns 11 and 12. Serial dilutions were performed by transferring 100μL from the wells of row A to the wells of row B and so forth, resulting in the concentrations shown in Figure-1. For each test well, 10μL of the respective inoculum was added (with turbidity equivalent to a 0.5 McFarland scale). For column 11, 10μL of saline solution was added (0.9%), and this served as a positive control. For column 12, 10 μL of inoculum was added, which served as a negative control. Finally, the plates were covered and incubated at 37°C for 24 h. After incubation, MIC was considered the lowest concentration of the tested material that inhibited the visible growth of the bacteria. MBCs were determined by subculturing the wells without visible growth (with concentrations ≥ MIC) on MHA plates. Inoculated agar plates were incubated at 37°C for 24 h. MBC was considered the lowest concentration that did not yield any bacterial growth on agar.

### Antibiotic modulation: experimental design, modeling, validation of the model, and optimization

Modulations of ampicillin and cefazoline were performed with ethanolic extracts (EEs) of grapefruit peel on the *E. coli* 2 (resistant to ampicillin) and *S. aureus* 1 strains (intermediate sensitivity to cefazolin) and *E. coli* 2 (resistant to cefazolin). The checkerboard method, commonly used for the determination of synergy between the antibiotics and natural antibacterial compounds, was used for the antibiotic modulation assay. The fractional inhibitory concentration (FIC) index was calculated, as described by Trabelsi *et al*. [29]. Briefly, the individual MICs of the two antibiotics (MIC-ATB) and the EE (MIC-extr) on the targeted strains were first determined using the microdilution method as described above. Then, the new MIC values (MIC′-ATB and MIC′-extr) were determined after combining the two substances. To assess the interaction between the antibiotic and the natural extract, the FIC was determined using the formula: *FIC*=*FICA*+*FICB*, with: 
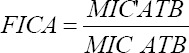
 and 
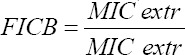
 The FIC index was interpreted as follows FIC ≤0.5, synergy; 0.5 ≤FIC ≤1, addition of effects; 1≤ FIC≤4, indifference and for FIC >4, Antagonism.

**Figure-2:**
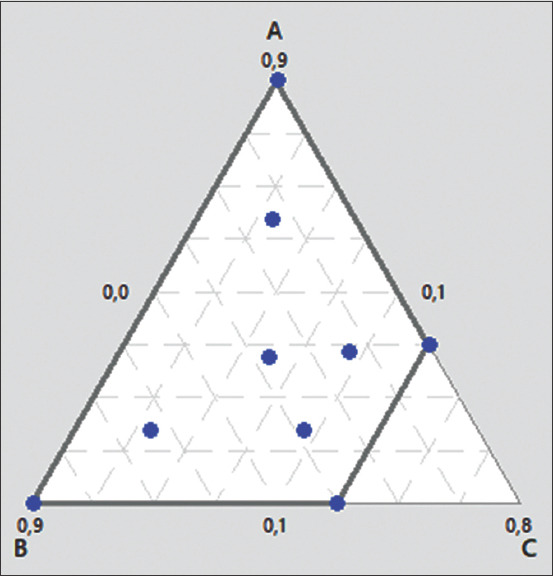
Simplex design plot in amounts, A=Cefazolin; B=Ethanolic extract of grapefruit peel, C=Distilled water.

Mixture design response surface methodology (MDRSM) with extreme vertices design was used to carry out the experiments to model and optimize the FIC index and the inhibition diameter of each combination. The independent variables (factors) were (A) concentration of antibiotic (cefazolin [Plan 1] or ampicillin [Plan 2]); (B) concentration of EE of grapefruit peel; and (C) distilled water. The intervals of these factors were 10% MIC to 90% MIC for both antibiotic and EE and 0%-80% for distilled water (Figure-2). The interval values of the factors were chosen considering the literature on the modulation of antibiotics with other plant extracts.

Mathematical models describing the relationships among the modulation-dependent variable and the independent variables in a second-order equation were developed [30].

Design-based experimental data were matched according to the following second-order polynomial equation:


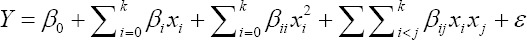


where *Y* is the response, *x*_i_ and *x*_j_ are the variables, *β*_0_ is the constant, *β*_i_ is the coefficient of the linear terms, *β*_ii_ is the coefficient of the quadratic terms, and *β*_ij_ is the coefficient of the interaction terms.

The coefficients of the models and statistical analysis (analysis of variance) were obtained using the Minitab version 18 software (Minitab, Ltd., Brandon Court, Unit E1-E2 Progress Way, Coventry, CV3 2TE, UK). The curves were plotted using Sigmaplot version 12.1 (Systat Software, Inc., 1735 Technology Drive, Suite 430, San Jose, CA 95110, USA).

Validating the models was obtained by calculating the absolute average deviation (AAD), the bias factor (*B*f), and the accuracy factor (*A*_f_) [30,31], which were obtained as follows:


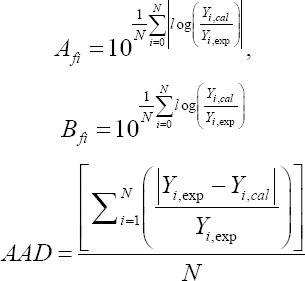


where *Y*_i,exp_ and *Y*_i,cal_ are, respectively, experimental and calculated responses and *N* is the number of experiments used in the calculation.

Each linear, interaction, and quadratic contribution of each factor were obtained as follows:

For linear terms,


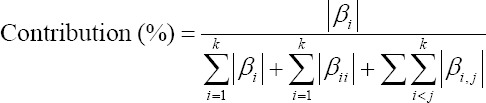


For quadratic terms,


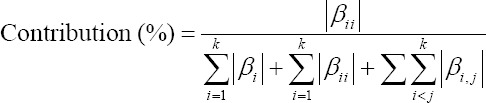


For interaction terms,


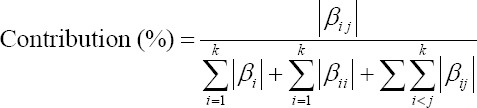


Finally, optimization was done using the software Minitab 18 (Minitab, Ltd.). The conditions fixed were to maximize the inhibition diameter and to minimize the FIC index.

### Statistical analysis

All experiments were carried out in triplicate. The inhibition diameters measured in the antibiotic sensitivity test were interpreted according to the Clinical and Laboratory Standards Institute [32]. Resistance R, Intermediate I, and Sensitive S interpretations were obtained automatically using algorithms written in Excel software [Microsoft Office 2016 MSO version 16.0.13628.20128(32 bits), USA]. The statistical significance was set at p≤0.05. A t test and principal component analysis (PCA) were carried out using XLSTAT 2020 (Addinsof Inc., New York, USA) statistical software. PCA was done to visualize the correlations between the MICs, MBCs, and the bacteria tested.

## Results and Discussion

### Sensitivity of bacteria to antibiotics

The sensitivity of the five bacteria used in this study (*E. coli* 1, *E. coli* 2, *S. aureus* 1, *S. aureus* ATCC 6538, and *E. faecalis*) to eight antibiotics was determined (Table-1) and the multidrug resistance (MDR) index of each bacterium was calculated. The standard strain, *S. aureus* ATCC 6538, was sensitive to all of the antibiotics tested (MDR=0), whereas the clinical *S. aureus* 1 strain was resistant to trimethoprim and cefazolin plus clavulanic acid (MDR=0.25). The phenotypic resistance of the *S. aureus* 1 clinical strain may have resulted from resistance acquired through exposure to various antimicrobials. In addition, the *E. coli* 1 and *E. coli* 2 strains were both resistant to ampicillin, amoxicillin, and trimethoprim, whereas only *E. coli* 1 was resistant to cefazoline and ceftriaxone. This result is consistent with that obtained by various authors on the resistance of clinical strains to antibiotics [33-35]. The problem of antibiotic resistance exists worldwide and affects all sectors that use antibiotics [34]. Various means have been implemented in recent years to provide effective solutions to this problem, and the studies carried out target bacteria that are resistant or not. The present study focused on evaluating the antibacterial properties of grapefruit peel extracts, a byproduct, and its green-synthetized AgNPs.

**Table-1:** Sensibility of bacteria to antibiotics.

Bacteria	AMP	AMC	TR	CZ	CTR	CIP	CAC	NIT
*Escherichia coli 1*	6.0±0.0 (R)	6.0±0.0 (R)	6.0±0.0 (R)	6.0±0.0 (R)	12.5±1.4 (R)	26.5±0.7(S)	21.0±0.0(S)	20.5±0.7(I)
*Escherichia coli 2*	6.0±0.0 (R)	6.0±0.0 (R)	6.0±0.0 (R)	19.0±1.4 (I)	24.0±2.8(S)	21.0±1.4(S)	21.0±0.0(S)	20.5±0.7(I)
*Staphylococcus aureus 1*	31.5±0.7 (S)	22.5±0.7 (S)	6.0±0.0 (R)	18±0.0 (I)	21.5±1.4(S)	23.5±2.1(S)	15.0±0.0 (R)	18.0±1.4 (I)
*Staphylococcus aureus* ATCC 6538	33.5±0.7 (S)	30.5±0.7 (S)	31.5±0.7 (S)	30.5±0.7 (S)	29.0±0.0(S)	26.0±1.4(S)	21.0±0.0(S)	23.5±0.7(S)
*Enterococcus faecalis*	29.0±0.0 (S)	22.0±1.4 (S)	23.0±2.8 (S)	30.5±2.1(S)	24.5±1.4(S)	25.5±2.1(S)	17.5±0.7 (I)	20.0±1.4 (I)

### Extract yield

The extraction of 30 g of grapefruit peel was carried out for 24 h at 25°C with continuous stirring in 270 mL of water and 270 mL of ethanol 80% (v/v). The volume yield was 96.2% (v/v) for the EE and 93.8% (v/v) for the AE. Interestingly, the highest yield of crude extract was obtained from the AE (9.41%; 2.823 g), whereas 7.84% (2.352 g) was obtained with the EE. The extraction performance depends on several factors, including the extraction method, time, the solvents, and the quality of the equipment used. The extraction yield varies greatly between studies and similarly in our study. Some researchers have obtained aqueous extraction yields higher than the ethanolic yield [36,37], whereas others have made opposite observations [38,39].

### Visual observation, UV–vis spectroscopy, and AgNPs diameter

**Figure-3:**
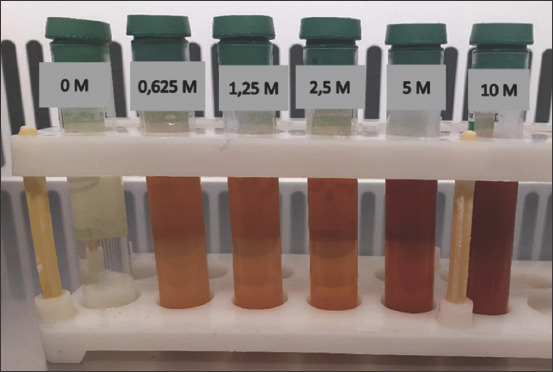
Coloration of solution after green-synthesis of silver nanoparticles with various concentrations of AgNO_3_.

**Figure-4:**
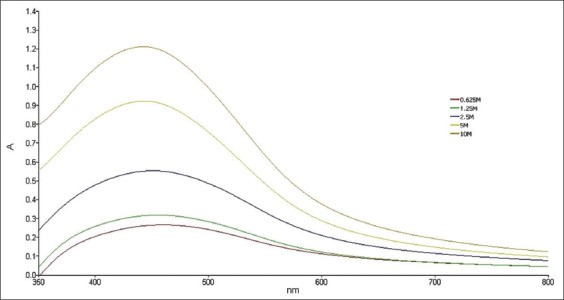
Ultraviolet-Vis of silver nanoparticles synthetized.

**Figure-5:**
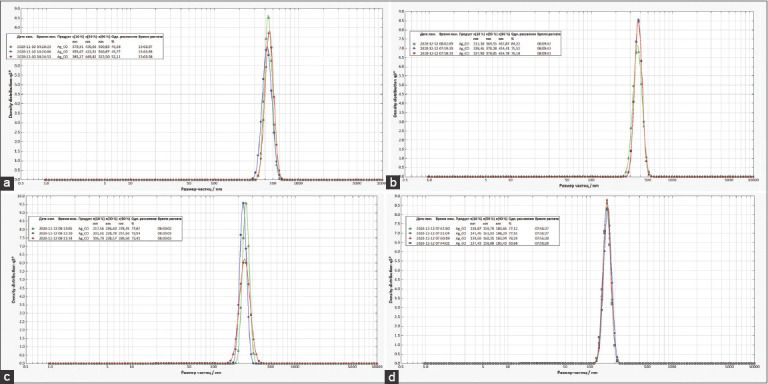
Diameter of particles and silver nanoparticles green synthetized with various concentrations of AgNO_3_, 10 Mm (a), 5 mM (b), 2.5 mM (c), and 1 mM (d).

In all experiments, the addition of grapefruit peal extract to an aqueous solution of silver nitrate led to a change in color of the solution from yellowish to reddish-brown with an accentuation of the color as the concentration of AgNO_3_ increased (Figure-3). It is well known that AgNPs exhibit a yellowish-brown color in an aqueous solution because of the excitation of surface plasmon vibrations in AgNPs [13]. AgNPs were synthesized at different concentrations of AgNO_3_ by keeping the volume of the aqueous grapefruit extract constant (1 mL + 9 mL of AgNO_3_), and all the solutions obtained were analyzed by UV–vis at 350–800 nm (Figure-4). We noticed that the curves of the UV–vis spectra had all the same tendencies, but the optical density increased with the increase in the AgNO_3_ concentration, thus confirming the visual observation. Some authors have suggested that the increase in density means that the quantity of AgNPs synthesized increases, whereas others concluded that it was the result of an increase in the size of the nanoparticles. This second hypothesis was verified in our study because, as shown in Figure-5, the diameters of the nanoparticles measured by PCCS varied significantly depending on the concentration of AgNO_3_. Indeed, we obtained average hydrodynamic particle diameters, 50× of 436.13 ± 13.56 nm (Figure-5a), 375.29±4.97 nm (Figure-5b), 238.01±8.96 nm (Figure-5c), and 160.5±1.74 nm (Figure-5d), respectively, with AgNO_3_ concentrations of 10, 5, 2.5, and 1 mM. Conventionally, nanoparticles are defined as particles having a diameter ranging from 0 to 100 nm [40]. However, recent studies have generated particles with sizes of 145 [41], 150 [42], 160 [11,12], 175 [43], and 180 nm [44] as AgNPs. That is why we considered silver particles of 160.5 nm size green-synthesized with 1 mM of AgNO_3_ as AgNPs in the remainder of the study. Although several methods, such as microwave irradiation, milling, or ultrasonication, are sometimes used to reduce the size of nanoparticles, they were not applied in this study to remain within the framework of strictly green synthesis. Moreover, concerning absorbances, the results were similar to data reported in the literature showing the optimal absorbance between 430 and 450 nm for AgNPs synthesized with various plant extracts with AgNO_3_ as a precursor [10-13]. In addition, we noticed in the UV–vis spectra that the curves obtained were all uniform, which shows a certain uniformity in the composition of the solutions. This observation was confirmed when measuring the diameters of the AgNPs and studying the distribution of the nanoparticles by PCCS. Overall, we conclude that, despite the relatively large size of the AgNPs synthesized in this study, grapefruit peel extract can contribute to the synthesis of AgNPs with good distribution and homogeneity.

### Antibacterial activity of grapefruits peel extracts and green-synthetized AgNPs

**Figure-6:**
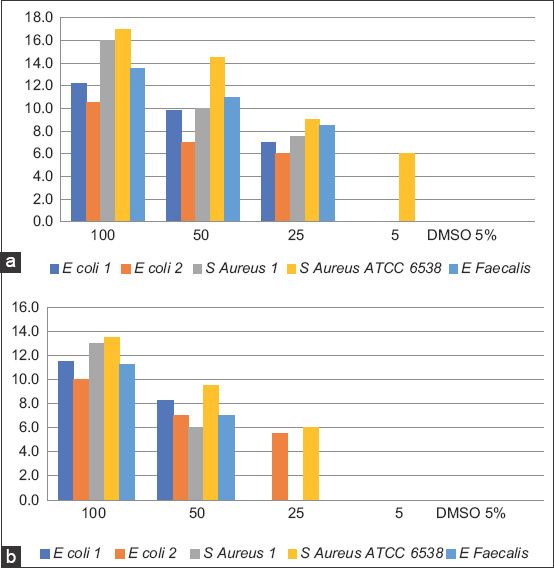
Results of the screening of the antimicrobial activity of the ethanolic (a) and aqueous (b) extracts of grapefruit peel.

**Figure-7:**
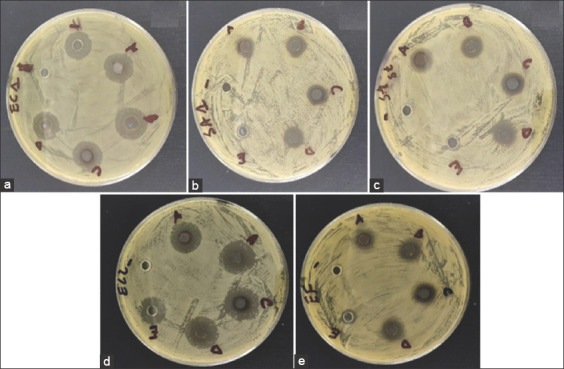
Antibacterial activity of silver particles and silver nanoparticles green-synthetized against *Escherichia coli* 1 (a), *Staphylococcus aureus* 1 (b), *S. aureus* ATCC 6538 (c) *E. coli* 2 (d), and *Enterococcus faecalis* (e).

Grapefruit peel ethanolic and AEs were evaluated for their antibacterial activity against five bacteria, including three Gram-positive bacteria (*S. aureus* 1, *S. aureus* ATCC 6538, and *E. faecalis*) and two strains of Gram-negative bacteria (*E. coli* 1 and *E. coli* 2) using the disk diffusion method. The results of the antimicrobial activity measured using the well diffusion method are shown in Figure-6 for the aqueous and EEs and in Figure-7 for the AgNPs. As expected, 5% DMSO as a negative control did not show an inhibition zone against any of the tested bacteria. The results revealed that the effects of both extracts (aqueous and ethanolic) were dose-dependent. Regardless of the bacterial strain tested and the type of extract used, the inhibition diameters decreased with a decrease in the concentration of the extract. Similar observations have been made in most studies investigating antibacterial properties, particularly of plant extracts, and antimicrobial compounds, more generally. Furthermore, no antibacterial activity was observed for concentrations ≥5 mg/mL of AE, whereas for the EE, an antibacterial effect was observed on the *S. aureus* ATCC 6538 strain. In addition, as shown in Figure-7, the synthesized AgNPs exhibited antibacterial activity on all bacteria tested with larger inhibition diameters on Gram-negative bacteria. This indicates that AgNPs are broad-spectrum antimicrobial agents. It is well known that AgNPs have a more bactericidal effect on Gram-negative bacteria than on Gram-positive bacteria. This may be due to the cell wall structure of Gram-negative bacteria, which differs from the structure of Gram-positive bacteria with a thin layer of peptidoglycan, the presence of a periplasm, and ease of exchange on the plasma membrane [45]. AgNPs have been used over the past decades to prevent and treat various diseases and are well known as antimicrobial agents because of their strong biocidal effect against microorganisms [45]. Their exact mechanism of action against bacteria is still largely unknown, although some researchers have suggested that their action on bacteria may result from their ability to adhere to the cytoplasmic membrane and cell wall to cause disruption and penetrate the cell [46], the formation of free radicals [45], the inactivation of proteins in the cell by silver ions [47], and the production of reactive oxygen species [45].

### MICs and MBCs of the extracts and green-synthetized AgNPs

The MIC and MBC of the ethanolic and AEs of the grapefruit peels, as well as those of the green-synthetized AgNPs on the five bacterial strains (*E. coli* 1, *E. coli* 2, *S. aureus* 1, *S. aureus* ATCC 6538, and *E. faecalis*) tested, are presented in Table-1. The MICs (expressed in mg/mL) of the AEs varied from 3.125 (*S. aureus* ATCC 6538) to 12.5 (*E. coli* 1 and *E. coli* 2), whereas the MICs of the EEs varied from 1.5625 (*S. aureus* 1, *S. aureus* ATCC 6538, and *E. faecalis*) to 6.25 (*E. coli* 1). After determining the MICs using the microdilution method, the MBCs were confirmed by streaking the contents of the wells having a concentration greater than the MIC on MHA and choosing the lowest concentration where no growth was observed as the MBC. The MBCs (mg/mL) of the AEs varied from 12.5 (*S. aureus* ATCC 6538) to 50 (*S. aureus* 1), whereas those of the EEs varied from 6.25 (*S. aureus* 1) to 25 (*E. coli* 1 and *E. faecalis*). A paired sample t test revealed that there was a significant difference between the MICs of the AEs and EEs (p=0.014). This difference in activity between the two extracts may be explained by the fact that some bioactive components of medicinal plants may differ in their solubility depending on the solvents used for preparation [48]. Otherwise, the results of this study are similar to those of Özogul *et al*. [19], Deng *et al*. [21], and Presentato *et al*. [49]. A large variation in the MIC of grapefruit peel extract demonstrated in several investigations may result from considerable variation in the extraction method, chemical constituents, and bacterial strains tested. Overall, grapefruit peel extracts were more effective against Gram-positive than against Gram-negative bacteria. The higher sensitivity of Gram-positive bacteria compared with Gram-negative bacteria may be explained by the differences in the composition of their cell walls. Gram-positive bacteria have an outer wall made essentially of peptidoglycan, which is an ineffective permeability barrier [50], whereas the outer phospholipid membrane of Gram-negative bacteria makes the outer layer impermeable to lipophilic solutes and constitutes a selective barrier to hydrophilic solutes.

**Figure-8:**
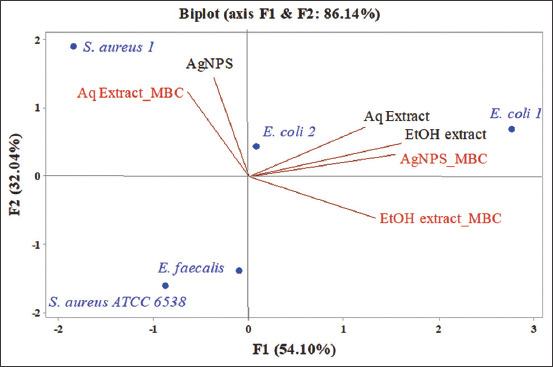
Principal component analysis of minimum inhibitory concentration and minimum bactericidal concentration of silver nanoparticles green-synthetized, aqueous, and ethanolic extract of grapefruit peel against *Escherichia coli*, *Staphylococcus aureus* and *Enterococcus faecalis*.

Although the nanoparticles synthesized in this study with 1 mM of AgNO_3_ were relatively large (160 nm), their MICs and MBCs were determined (Table-1). The MICs of AgNPs ranged from 3.125 mg/mL (*E. faecalis*) to 50 mg/mL (*S. aureus* 1). Surprisingly, the MIC of the AgNPs on *S. aureus* ATCC 6538 (6.25 mg/mL) was far lower compared with that of the clinical strain *S. aureus* 1, although the two strains belong to the same species. This ­observation raises new questions, including whether clinical strains, because of their history in different habitats and their exposure to various substances, can adapt to antimicrobial agents and even nanoparticles. On the one hand, Anuj *et al*. [51] revealed that the size of the bacteria had an influence on the effectiveness of the antimicrobials tested and microbes with a large surface area are more difficult to treat than small size microbes. Since the diameter of staphylococci generally varies from 0.5 to 1.5 mm, it may be necessary to conduct additional studies to assess the size of the two strains of *S aureus* (*S. cereus* 1 and *S. aureus* ATCC 6538) used in this study. However, the hypothesis of acquired resistance appears to be a more likely explanation for this observation. Indeed, Valentin *et al*. [52] revealed that exposure of bacteria to AgNPs can lead to mutations endowing these microbes with genotypic resistance traits that can be transmitted between bacteria similar to conventional antibiotics. Therefore, this phenomenon should be carefully monitored, and it suggests that nanoparticle antimicrobials should only be used when needed to maintain their effectiveness in treating infections [52]. Furthermore, AgNPs also exhibited bactericidal activity against *S. aureus* ATCC 6538, *E. faecalis*, and *E. coli* 1, whereas the MBC of *E. coli* 2 and *S. aureus* 1 could not be determined because they were superior at the initial AgNP concentration that was prepared (100 mg/mL). To visualize the association between the microbial species tested in this study, the MICs and MBCs of the aqueous and EEs, a PCA was performed. Figure-8 shows the distribution of the tested bacteria, MICs, and MBCs in an F1 × F2 system. The strains *S. aureus* ATCC 6538 and *E. faecalis* were relatively similar with respect to their sensitivity to the antimicrobials tested. The same similarity was verified in the two strains of *E. coli* (1 and 2) tested, whereas the *S. aureus* 1 strain appeared to exhibit the opposite result from the other bacteria. Moreover, the MICs of the aqueous and EEs were strongly correlated with the MBC of AgNPs, which was expected because the quantities of AgNPs necessary to have a significant antibacterial effect are always very low, whereas the quantities of plant extract necessary to obtain a similar antimicrobial effect depends on the plant used and are generally greater. Overall, the MIC and MBC results observed in this study suggest that grapefruit peels and its green-synthesized AgNPs can be exploited for their antibacterial properties in several sectors, including pharmaceuticals (for the development of new antimicrobials), medicine (for the treatment of bacterial infections), agriculture, and animal breeding. However, more studies are needed to determine the exact composition of the grapefruit peel extracts and to assess their toxicity and safety.

### Modulation of cefazolin and ampicillin with grapefruit peel extract

**Figure-9:**
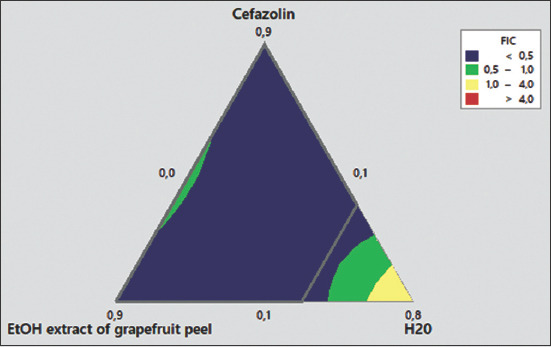
Response surface of fractional inhibitory concentration (FIC) index as function of proportion of cefazolin (A), ethanolic extract of grapefruit peel (B) and water (C) on *Staphylococcus cereus* 1. FIC=0.0831A + 0.2469B + 0.507C + 0.227AB −0.85AC −0.42BC.

**Figure-10:**
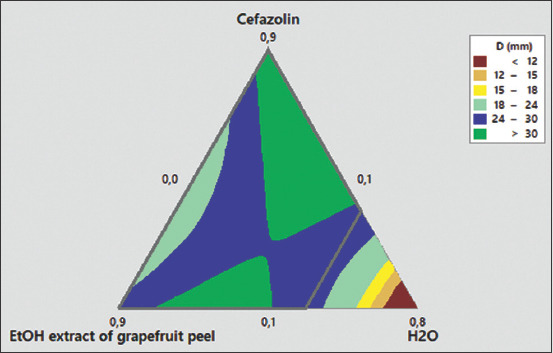
Response surface of inhibition diameter of *Staphylococcus* *cereus* 1 as function of Proportion of cefazolin (A), ethanolic extract of grapefruit peel (B) and water (CD (mm) =40.5A + 31.25B −24C −65.5AB + 94.4AC +99BC.

The use of combination therapy has been suggested as a new approach to improve the efficacy of antimicrobial agents by screening crude extracts from medicinal plants with good indications for use in combination with antibiotics [53]. In this study, the EE of grapefruit peel was used for the modulation of antibiotics since it demonstrated the best antibacterial effect. The modulation of ampicillin was tested on the *E. coli* 2 strain, which was resistant to ampicillin, whereas the modulation of cefazoline was tested on the *S. aureus* 1 strain, which showed intermediate sensitivity to this antibiotic, and on *E. coli* 2, which was resistant to cefazoline (Table-2). The MDRSM with extreme vertices design was used to assess and predict the FIC index and inhibition diameters resulting from each formulation on the bacteria tested. The three factors for this plan were the proportions of antibiotic (A), the EE of grapefruit peel (B), and distilled water (C), which varied from 0.1 MIC to 0.9 MIC for the first two factors and from 0 to 0.5 for distilled water. The role of distilled water was to complete the mixture to have the desired concentration of the antibacterial. The results are shown in Table-3. The combination of ampicillin and EE was completely indifferent to the resistant *E. coli* 2 strain. However, the combination of cefazolin and EE showed a synergistic effect and reduced MIC on *E. coli* 2 and *S. aureus* 2. Regarding the *E. coli* 2 strain, the results observed for the mixing plan indicated that there was a synergy between cefazolin and the ethanolic grapefruit extract for concentrations of cefazolin ≥0.375 MIC combined with concentrations of the extracts ≥0.1 MIC. This result was closely correlated with the inhibition diameters obtained. In addition, for *S. aureus* 2, the synergistic action of the EE and cefazolin was modeled according to the monitoring of the diameters of inhibition (D) and the FIC index. The two models (D and FIC) were validated on the basis of the coefficient of determination R^2^ (0.95 and 0.89), AAD (0.005 and 0.082), the *B*f (1.005 and 1.003), and the *A*_f_ (1.000 and 1.083). As shown in Figures-9 and 10, the resulting contour plots showed that there is a synergistic action (FIC ≤0.5) for most of the formulations made between the limits of the proportions fixed in the study. However, when the proportion of water was very small or slightly high, additional effects of the two antimicrobials were observed (0.5 ≤ FIC ≤1). In addition, the very proportion of water-induced an indifference between cefazolin and the ethanolic grapefruit peel extract (1 ≤ FIC ≤4). Nevertheless, despite the formulation preparation between the limits of this study, no antagonistic effect between the two substances was observed (FIC ≥4). Although a synergistic effect was observed in all formulations, optimization of the synergistic effect by minimizing the FIC and maximizing the diameters of inhibition revealed that the optimal synergistic effect was obtained with a mixture having proportions of 49% of cefazolin (0.49 MIC), 22% of ethanolic grapefruit peels extract (0.22 MIC), and 29% distilled water. Therefore, our results suggest that EEs of grapefruit peel may reduce the MIC of cefazolin on bacteria, including resistant strains. These extracts may act as a substrate to circumvent or inhibit the efflux pump contained in the *S. aureus* and *E. coli* strains. Similar observations have been reported with various plants; however, to the best of our knowledge, no similar study has been performed to date with grapefruit peel. The potent antibiotic potentiation activity of grapefruit peel suggests a use for extending the life of older antibiotics that have lost efficacy in fighting bacterial infections.

**Table-2:** MIC and MBC of silver nanoparticles green-synthetized, aqueous and ethanolic extract of grapefruit peel against *Escherichia* coli, *Staphylococcus* *aureus,* and *Escherichia* faecalis.

*B*acteria	MIC (mg/mL)				MBC (mg/mL)			
	
	Aq extract	EtOH extract	CZ	AgNPs	Aq extract	EtOH extract	CZ	AgNPs
*Escherichia coli 1*	12.5	6.25	ND	0.025	25	25	ND	0.5
*Escherichia coli 2*	12.5	3.125	0.025	0.025	25	12.5	0.1	ND
*Staphylococcus aureus 1*	6.25	1.5625	0.0125	0.05	50	6.25	0.1	ND
*Staphylococcus aureus* ATCC 6538	3.125	1.5625	ND	0.00625	12.5	12.5	ND	0.025
*Enterococcus faecalis*	6.25	1.5625	ND	0.003125	25	25	ND	0.0125

AgNPs=Silver nanoparticles, MIC=Minimum inhibitory concentration, MBC=Minimum bactericidal concentration

**Table-3:** Modulation of cefazolin with ethanolic extract of grapefruit peel.

Trials	Proportion			Initial concentration			Volume (mL)			*Escherichia coli* 2		*Staphylococcus aureus*			
				
	CZ	EtOH extract	H_2_O	ATB (µg/mL)	EtOH extract (mg/mL)	H_2_O	ATB	EXTR	H_2_O	D (mm)	FIC	D exp (mm)	D cal (mm)	FIC exp	FIC cal
1	0.9	0.1	0	MIC	MIC	-	4.500	0.500	0.000	15.00	0.08	34	33.68	0.13	0.12
2	0.2375	0.6375	0.125	MIC	MIC	-	1.188	3.188	0.625	0	ND	28	27.32	0.22	0.22
3	0.1	0.4	0.5	MIC	MIC	-	0.500	2.000	2.500	0	ND	26	26.45	0.25	0.24
4	0.375	0.375	0.25	MIC	MIC	-	1.875	1.875	1.250	0	ND	30	29.83	0.19	0.16
5	0.3875	0.2375	0.375	MIC	MIC	-	1.938	1.188	1.875	8	0.17	30	30.62	0.16	0.14
6	0.6375	0.2375	0.125	MIC	MIC	-	3.188	1.188	0.625	14	0.20	30	30.79	0.11	0.13
7	0.1	0.9	0	MIC	MIC	-	0.500	4.500	0.000	0	ND	26	26.28	0.25	0.25
8	0.4	0.1	0.5	MIC	MIC	-	2.000	0.500	2.500	10	0.10	29	28.54	0.13	0.13
9	0.2375	0.3875	0.375	MIC	MIC	-	1.188	1.938	1.875	0	ND	30	29.49	0.16	0.19

MIC=Minimum inhibitory concentration, MBC=Minimum bactericidal concentration, FIC=Fractional inhibitory concentration

## Conclusion

The results of this study suggest that grapefruit peels, a byproduct, possess compounds with antibacterial properties that may be used in the fight against antibiotic resistance and in the synthesis of new drugs for the treatment of bacterial diseases. Nevertheless, further studies are needed to evaluate the mechanisms of action and toxicity of these extracts. In addition, the AgNPs, although relatively large in size, were also successfully synthesized from grapefruit peel through a simple green and eco-friendly route. They exhibited antibacterial activity against both Gram-positive and Gram-negative bacteria with a greater effect on the latter. Similarly, further studies should be carried out to optimize the synthesis process of AgNPs with grapefruit peels by evaluating the parameters affecting size and to establish toxicity profiles.

## Authors’ Contributions

MMJA and AKLD: Conceptualized and designed the study. MMJA and ANS conducted the laboratory experiments. MMJA, AKLD, IVP, MR, and MSD: Wrote the first manuscript draft, edited, and revised the final version of the manuscript. All authors have read and approved the final manuscript.
